# Analytical Purity Determinations of Universal Food-Spice *Curcuma longa* through a Q*b*D Validated HPLC Approach with Critical Parametric Predictors and Operable-Design’s Monte Carlo Simulations: Analysis of Extracts, Forced-Degradants, and Capsules and Tablets-Based Pharmaceutical Dosage Forms

**DOI:** 10.3390/foods12051010

**Published:** 2023-02-27

**Authors:** Hamdoon A. Mohammed, Dhafer S. Alsahabi, Amira M. Hegazy, Riaz A. Khan, Adel M. Ahmed

**Affiliations:** 1 Department of Medicinal Chemistry and Pharmacognosy, College of Pharmacy, Qassim University, Qassim 51452, Saudi Arabia; 2 Department of Pharmacognosy and Medicinal Plants, Faculty of Pharmacy, Al-Azhar University, Cairo 11371, Egypt; 3 PharmD Graduate, College of Pharmacy, Qassim University, Qassim 51452, Saudi Arabia; 4 Pharmaceutical Analytical Chemistry Department, Faculty of Pharmacy, Beni-Suef University, Beni-Suef 62574, Egypt; 5 Department of Pharmaceutical Analytical Chemistry, Faculty of Pharmacy, South Valley University, Qena 83523, Egypt

**Keywords:** drug screening, chemical stability, quality by design, curcuminoids, full factorial design, Monte Carlo simulations, critical method parameters, critical method attributes, method operable design region, Plackett–Burman design

## Abstract

Applications of analytical quality by design (Q*b*D) approach for developing HPLC (High Performance Liquid Chromatography) methods for food components assays, and separations of complex natural product mixtures, are still limited. The current study developed and validated, for the first time, a stability-indicating HPLC method for simultaneous determinations of curcuminoids in *Curcuma longa* extracts, tablets, capsules, and curcuminoids’ forced degradants under different experimental conditions. Towards separation strategy, critical method parameters (CMPs) were defined as the mobile phase solvents’ percent-ratio, the pH of the mobile phase, and the stationary-phase column temperature, while the peaks resolution, retention time, and the number of theoretical plates were recognized as the critical method attributes (CMAs). Factorial experimental designs were used for method development, validation, and robustness evaluation of the procedure. The Monte Carlo simulation evaluated the developing method’s operability, and that ensured the concurrent detections of curcuminoids in natural extracts, commercial-grade pharmaceutical dosage-forms, and the forced degradants of the curcuminoids in a single mixture. The optimum separations were accomplished using the mobile phase, consisting of an acetonitrile–phosphate buffer (54:46 *v*/*v*, 0.1 mM) with 1.0 mL/min flow rate, 33 °C column temperature, and 385 nm wavelength for UV (Ultra Violet) spectral detections. The method is specific, linear (R2 ≥ 0.999), precise (% RSD < 1.67%), and accurate (% recovery 98.76–99.89%), with LOD (Limit of Detection) and LOQ (Limit of Quantitation) at 0.024 and 0.075 µg/mL for the curcumin, 0.0105 µg/mL and 0.319 µg/mL for demethoxycurcumin, and 0.335 µg/mL and 1.015 µg/mL for the bisdemethoxycurcumin, respectively. The method is compatible, robust, precise, reproducible, and accurately quantifies the composition of the analyte mixture. It exemplifies the use of the Q*b*D approach in acquiring design details for developing an improved analytical detection and quantification method.

## 1. Introduction

**Figure 1:**
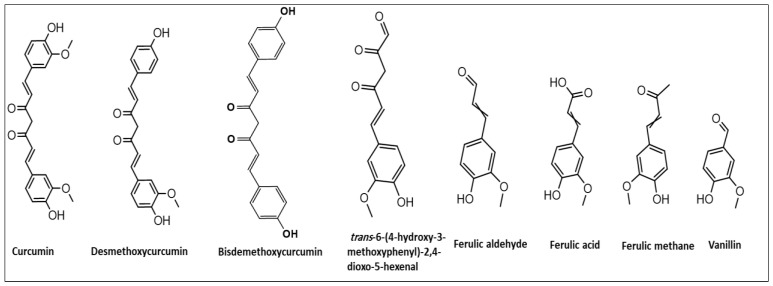
Chemical structure of curcuminoids, and their common degradants.

The universal spice turmeric, botanically named *Curcuma longa*, family Zingiberaceae, is well known as the curry-coloring spice. It is frequently used as the commonest food-additive throughout the globe. Its use is much prevalent in the oriental worlds of India, China, Indo-China, and the Mediterranean regions. The dried root powder of the plant is commercially available at groceries and spice shops. The herb powder is consumed for several culinary purposes to induce color, spiciness, taste, fragrance, and flavor [1]. The edible herbal powder possesses several biological activities, i.e., anticancer, antimicrobial, antiviral, anti-inflammatory, and wound-healing [2,3,4,5,6,7,8]. Curcuminoids are the main constituents in root powder and are considered responsible for the pharmacological efficacy of the powder. The primary constituents are curcumin, demethoxycurcumin, and bisdemethoxycurcumin [9], while different structural degradants have also been identified (Figure 1).

The structural similarities among the phytoconstituents, and the sensitivity of the *Curcuma* powder exposure to light, moisture, and other unnatural and forced conditions, including the powder’s proneness to degradation over time, lead to compositional changes [10]. This situation has raised issues about the purity, efficiency, and overall quality of the powder for human consumption. Several studies related to analytical purity, composition profile of the powder, and curcumin-based commercial products containing curcumin and other curcuminoids, including their degradants, have led to various investigations for developing isolation–purification, impurity profiling, structural analyses, componential assays, and contents-tracing through spectro-analytical and chromatographic techniques development. A number of reports on HPLC (High Performance Liquid Chromatography) analyses of the *Curcuma* powder under different experimental conditions, including bio-analytical procedures, instrumental backgrounds, and mobile and stationary chromatographic phases are known [11,12,13,14,15,16,17,18,19,20,21]. The current approach aimed to develop a Q*b*D (Quality by Design) analytical purity analysis method with different factors playing a part in the separation of compounds of the *Curcuma* powder mixture. These critical factors in conjunction with simulations of the resolution phenomenon and methods design approach indicators were employed to arrive at an optimally resolved chromatographic analysis of the *Curcuma* powder components, and their degradants. The current method also aimed to analyze the purity conditions of the commercially available *Curcuma* powder formulations of tablets and capsules.

Nonetheless, a plethora of work has recommended the HPLC technique with UV (Ultra Violet) spectrum-based detections, as one of the best-suited analytical methods for analyzing the high contents of curcuminoids in the curcumin powder, and its different formulation forms. However, certain limitations related to a comparatively longer time of analysis as a consequence of longer retention time, the need for a complicated gradient-elution-based mobile phase solvent system, higher margins in LOD (limit of detection), and high mobile-phase flow rates [22,23,24], have necessitated improvements in the HPLC-based analytical methodology. Another analytical target needing attention is the simultaneous detection of curcuminoid products, namely, curcumin, demethoxycurcumin, and bisdemethoxycurcumin. The detection of curcuminoids degradants, i.e., ferulic acid, ferulic aldehyde, ferulic methane, and vanilla, are also some of the most common degradation products needing attention.

An approach was developed to overcome the separations, and detection pitfalls underplaying in the curcumin’s components resolution, retention time, and possibilities in the gradient-based mobile phase’s componential variability for its effective and optimum flow rate, as well as temperature, and pH fluctuations. The critical method parameters (CMPs), i.e., the resolutions among the components, their retention times, the number of involved theoretical plates, and the method’s operable designs, are some of the parameters that were considered as the factors in the method development. For experimental set-up, for example, pH, temperature effectiveness in the stationary phases during separations, and the mobile phase components’ variability were selected to find an optimized region of maximum operability conditions for the analytical method. The method development was formulated to achieve the analytical target profiles of the curcuminoid products and their degradants in the separation. Monte Carlo simulations validated the critical parameters for the developing method and its design factors. It paved the way for the Q*b*D approach to a validated method development, which was needed for making the procedure capable of resolving the closely eluted components, and for better quantitation in the given curcumin powder of fresh and degraded samples. The analytical target profiling of these separations of the curcuminoid products and their degradants was achieved through the designated HPLC-based separations. It also incorporated the critical method attributes (CMAs), leading to the identification of the associated CMPs responsible for better separations, through maneuverability in the resolution, retention times, and the conditions of pH, as well as the flow-rate and polarity controls through the composition of the mobile phase. The adeptness to the variability of the parameters on separation, the method’s working feasibility, separation out-reach of the method without losing the analytical targets, precision, and robustness were addressed and confirmed through the quality risk assessment, a key component of the Q*b*D approach. The approach was traversed through the CMPs. The Method Operable Design Region (MODR) factors for the risk assessment of the quality components were also investigated. The MODR approach was used for the development of an RP-HPLC (Reverse Phase High-Performance Liquid Chromatography) method for the simultaneous determinations of the curcuminoids, and their degradants. The starting step was to examine, and extrapolate the prior knowledge about the analytical behavior of these products, and define the analytical target profile (ATP) of the products under investigation. The quality risk-assessment tools were used to define the CMPs through the application of the design of experiment (DoE) way, which resulted in defining the MODR in the ensuing analysis for developing the analytical separation, and detection, with clear limits of detections. The robustness of the method was achieved with a well-defined design space, together with a viable analytical procedure to analyze the curcuminoids, their degradants in the commercial powders, different dosage forms, and other pharmaceutical formulations, including the nutraceutical/food-additive samples.

## 2. Materials and Methods

### 2.1. Chemicals and Reagents

All reagents used in this study were of analytical grade. Acetonitrile (ACN) and methanol (MeOH) were purchased from Fisher Scientific; Milli-Q water used to prepare buffer solutions was obtained by a Millipore^®^ purification system (Bedford, MA, USA). K_2_HPO_4_, phosphoric acid, sodium acetate, and acetic acid were purchased from Merck (Darmstadt, Germany); turmeric rhizome was purchased from the herbal drug market. Standards of curcumin, demethoxycurcumin, and bisdemethoxycurcumin (>98% in purity) were purchased from Sigma-Aldrich (St. Louis, MO, USA).

### 2.2. Extraction of Curcuma longa Constituents

High-grade *Curcuma longa* was purchased from the local market as rhizomes and reduced to a fine powder using a mechanical device. The extraction procedure was conducted to measure the effect of the solvent on the chromatographic performance of the analysis method. Acetone and ethanol (100 mL) were selected separately for extractions of two sets of 1.0 g *Curcuma longa* powder for 20 min, using an ultrasound bath (40 kHz and 135 W, model Max Clean 1400, Unique, São Paulo, Brazil). After completing the volume up to the mark with the mobile phase, 2.0 mL was diluted with the mobile phase into a 10 mL volumetric flask. The samples were then filtrated through a 0.45 um nylon syringe (Sinergia Cientifica, Campinas, Brazil), and packed into HPLC vials before injection into the HPLC system.

### 2.3. Preparation of Stock Solutions

The methanolic stock solutions of curcumin, demethoxycurcumin, and bisdemethoxycurcumin (10.0 mgmL^−1^) were prepared separately in methanol, and the standard working solutions were prepared through dilution with the mobile phase. Stock solutions were stored in an amber flask at −40 °C until chromatographic analysis.

### 2.4. Preparation of Sample Solution

#### 2.4.1. Curcumin Capsules

Five capsules (Solgar^®^ (Leonia, NJ, USA) containing full spectrum curcumin as soft gel capsules containing 48 mg of curcuminoids) were accurately weighed, and mixed with 20 mL of methanol in a 100 mL volumetric flask. The mixture was sonicated for 20 min, and after cooling, the solution was completed to the volumetric mark with the mobile phase. A total of 2.0 mL from the solution was diluted with the mobile phase into a 100 mL volumetric flask. The sample was filtered through a 0.45-µm before its injection into the HPLC system.

#### 2.4.2. Curcumin Tablets

Five tablets (Longvida^®^, Noblesville, IN, USA) with optimized curcumin containing 1000 mg of turmeric) were accurately weighed, and finely powdered. 1.0 gm of powder was weighed and transferred to a 100 mL volumetric flask, to which 20 mL of methanol was added. The mixture was sonicated for 20 min, and after cooling, the solution was completed up to the flask’s mark with the mobile phase. A total of 2.0 mL of the solution was placed in a 10 mL volumetric flask and diluted with the mobile phase. The solution was finally filtered using a 0.45 µm membrane filter before its injection into the HPLC system.

### 2.5. Chromatographic Conditions

Chromatographic analyses were performed on an Alliance 2685/2489 separation system (Waters, Milford, CT, USA). Chromatographic separations were achieved using a Waters Symmetry^®^ C18 column {150 mm × 4.6 mm, 5 μM (meter) particle size}. The appropriate wavelength for HPLC analysis of the curcuminoids was selected using Varian Cary 50 Spectrophotometer which provided the full UV-VIS (Ultra Violet-Visible) spectra (200–800 nm). Based on that, 385 nm was selected as the detection wavelength, and the injection volume was kept at 10 µL. The final selected working condition, where the mobile phase consisted of a mixture of ACN-K_2_HPO_4_ with pH adjusted 2.7, with a 54:46, *v*/*v* ratio. The flow rate was set at 1.0 mL min^−1^, and the column temperature was kept at 33 °C.

### 2.6. Forced Degradation Studies

#### 2.6.1. Acid and Base Degradations

The acid and base degradations were performed by transferring 1.0 mL of curcuminoids stock solution (1.0 mg mL^−1^) to a 10.0 mL amber volumetric flask, and diluting with 1 mL of 1.0 M HCl, and 1 mL of 1.0 N NaOH (separately). The flasks were placed at 80 °C for 2 h in the dark. After that, the solution was cooled, neutralized with 1.0 mL of 1.0 N NaOH, or 1.0 mL of 1.0 N HCl, diluted with the mobile phase, filtered through a 0.45 µm syringe filter, and injected into the HPLC system.

#### 2.6.2. Sunlight-Exposed Degradation

A 1.0 mL of the stock solution (1.0 mg mL^−1^) was placed with 1.0 mL distilled water in a 10 mL transparent volumetric flask. The flask was placed under sunlight for 6 h. After that, the solution was cooled and filtered through a 0.45 µm syringe filter, before its injection into the HPLC system.

#### 2.6.3. Oxidative Degradation

Oxidative degradation was performed by mixing 1.0 mL of the stock solution (1.0 mg mL^−1^) with 5.0 mL of hydrogen peroxide 5% solution. The solution was kept at RT (Room Temperature) in the dark for 4 h. After that, the solution was filtered through a 0.45 µm syringe filter, before its injection into the HPLC system.

#### 2.6.4. UV Irradiation Degradation

A total of 1.0 mL of curcuminoids stock solution (1.0 mg mL^−1^) in a 10 mL transparent volumetric flask was exposed to UV light (365 nm) in a photo-stability chamber for 4 h. After that, the solution was filtered through a 0.45 µm syringe filter before its injection into the HPLC system.

#### 2.6.5. Thermal Degradation

Curcuminoids powder was stored in an oven at 80 °C for 2.0 h to study dry-heat degradation. After that, the solution (1.0 mg mL^−1^) was prepared and diluted before its injection into the HPLC system.

#### 2.6.6. Validation of the Method

Validation studies were conducted using the optimized assay conditions according to ICH guidelines Q2 (R1) concerning linearity, detection limit, quantitation limit, accuracy, precision, and robustness.

### 2.7. Software

Empower-2 software was used for monitoring the HPLC signals. Design-Expert ^®^ 8.0.7.1 (Stat-Ease Inc., Minneapolis, MN, USA) was used for experimental design, data analysis, and calculations of desirability functions. Companion software (M/s Minitab Inc., State College, PA, USA) was used to perform Monte Carlo simulations.

## 3. Results and Discussion

### 3.1. Curcumin, and Curcuminoids’ Physico-Chemical Properties, and Elution Profile

Curcuminoids are polyphenolic compounds composed of two differently placed *ortho*-methoxyphenyl moieties connected by an α,β-unsaturated-β-diketone structural part (Figure 1). At the neutral pH and RT, the curcuminoids are water-insoluble, but they exhibit solubility under alkaline conditions. The products are relatively unstable and degrade rapidly upon exposure to sunlight as well as at neutral and alkaline conditions. However, the products are comparatively more stable under acidic conditions [25]. The environmental, naturally initiated, and forced-degradation products were identified as vanillin, ferulic acid, ferulic aldehyde, and ferulic (feruloyl) methane, together with auto-oxidized dicyclopentadiene as the degradants in the curcuminoids powder. The relative concentrations of these degradants differed according to the conditions, and durations of the conditions of pH and temperature, as also observed earlier [26]. The lipophilic nature, the levels of lipophilicity, solubility behavior, partition coefficients of the products, and degradants played an important part in the separation of these components in the powdery mixture analysis. A better understanding of the retention times of the products was assessed by the MarvinSketch^®^ software *v* 20.11.0. (Marvin (chemaxon.com), which predicted the lipophilicity, log *P*, and distribution coefficients, log *D*, of the curcuminoid products. The curcumin showed log *P* at 4.12, log *D* at *3.76*, the demethoxycurcumin’s log *P* at 4.28, and log *D* at 3.92, while the bisdemethoxycurcumin’s log *P* was predicted to be 4.44, and log *D* was predicted as 4.44, which suggested the elution order of these products from the RP-HPLC, wherein the bisdemethoxycurcumin suggested to be eluted first, followed by demethoxycurcumin, and lastly the curcumin. The predicted values of log *P* and log *D* for the expected degradations ranged between 1.18 and 2.14, and 1.22 and 2.14, respectively, and it was indicated that the low polarity degradation products would elute before the curcuminoid products [27]. As mentioned, the acidic pH is better at solubilizing, and is suitable for best separation on the RP-HPLC column owing to the higher pKa values; e.g., for curcuminoids, it ranged from 8.38 to 10.41. Furthermore, a literature survey revealed that pH 3.0 was frequently used for the separation of curcuminoids [24,28]. Henceforth, a range of pH between 2.5 and 3.5 was chosen for further investigations. Preliminary experimentations on ACN, and methanol, as an organic modifier of the mobile phase, showed better results with ACN. Potassium phosphate was tested as a buffer, and a 10 mM concentration was selected for the separation purpose. The influence of the maximum wavelength absorbance and mobile phase flow rate was examined, and the best results were obtained at a 1.0 mL min^−1^ flow rate with 385 nm wavelength for detection.

### 3.2. Analytical Target Profiling

The analytical target profile (ATP) was developed and validated to be a robust, efficient, and stability-indicating method, with accuracy > 99%, and a precision limit <2% for the simultaneous separations of the curcuminoids (RS ≥ 1.2), with the shortest possible analysis time in the run-of of the RP-HPLC during the study. The CMAs were analyzed for material resolution between the curcumin and demethoxycurcumin, together with separations between the demethoxycurcumin and bisdemethoxycurcumin, respectively, as denoted by resolution 1 (RS1) and RS2. The retention time (R_T_) of curcumin (R_T_C), the retention time of the demethoxycurcumin (R_T-DMCMN_), and the retention time of bisdemethoxycurcumin (R_T-BDMCMN_), as well as the number of theoretical plates for curcumin separation (TP_CMN_), and the number of theoretical plates required for demethoxycurcumin (TP_DMCMN_) and bisdemethoxycurcumin (TP_BDMCMN_) separations, were all considered [16]. For the beforehand test, the fallibility of these CMAs and CMPs was chosen for the RP-HPLC run, and a quality risk assessment (QRA) was performed on the parameters. The QRA work helped to ensure the achievement of the intended analytical target profile of well-resolved conditions for the HPLC run, with proper separations of the peaks, and the feasibly separated retention times, wherein the supportive roles of the mobile phases were also envisioned. These requisites were validated through the QbD approach by predicting the probable fallacy of the method in any of the domains of these parameters to achieve the superior resolution, as the method was intended to achieve [29].

**Figure 2:**
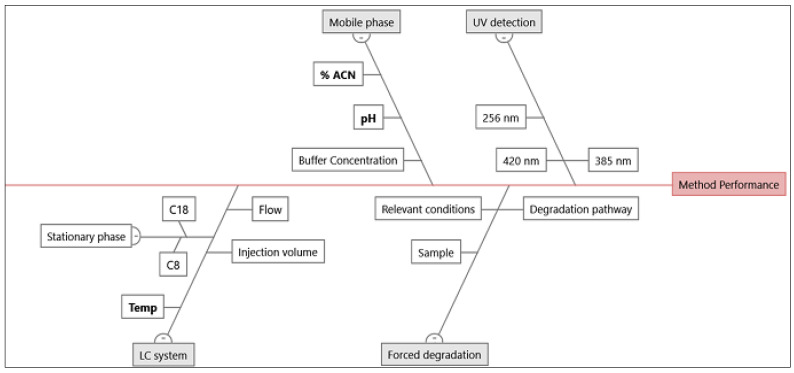
Ishikawa diagram showing the factors and parameters (boldface) affecting the HPLC separation.

To identify the risk associated with the approach involving the CMPs in achieving the target analytical profile, an Ishikawa diagram (Figure 2) was developed [30]. It was determined that the pH of the mobile phase, column temperature, and the gradient mobile phase’s composition as the ratio of the organic solvent were among the critical parameters supporting the requisite. The DoE (Design of Experiment) methodologies were employed to study the suggested parameters (Table 1). These parameters, with their settings of the level of operation, were selected for the QRA.

A *2^3^* full fractional design (FFD) with a total of eight experiments and three center points was planned and conducted, wherein each run was repeated in triplicate. All the runs were randomized to avoid systematic error [31]. The design expert software was used to analyze the obtained data (Table S1).

The ANOVA was performed, and the results (Table 2) showed that all *F*-values were large enough, which confirmed that the models were significant. The obtained *p*-values (< 0.0001) indicated that the models explained a significant portion of the variability. The adj. R^2^ (adjusted R^−^squares) were higher than 0.987, and that indicated that the experimental data had a good fit with the second-order polynomial, and the grouping variables were approximately 98.7% of the variation observed. The adequate (adeq.) precision values were greater than 4, and therefore the models were relevant for the separation process. The coefficient of variation (CV) for all the models was less than 10%, thereby indicating feasible and good model reproducibility. The regression’s lack of fit was determined by performing an *F*-test, and a good fit was assumed for all models, as the *p*-value was >0.0779 (*p*-value for lack of fit testing need be >0.05, non-significant).

### 3.3. Method’s Risk Assessments

The pH suggested a positive effect on the retention times of all the curcuminoids, while the column temperature and percentage of ACN had negative effects. The column temperature exerted a positive effect on the resolutions (RS_1_, RS_2_), while the buffer pH had negative effects. The number of theoretical plates was suggested to negatively affected the resolutions and retention times of all the curcuminoids (Table S2).

An optimization step to gauge the predictability of the experimental region of the CMPs was run using Derringer’s desirability functions. The best value for each parameter was optimized to find the desirable chromatographic conditions [32].

**Figure 3:**
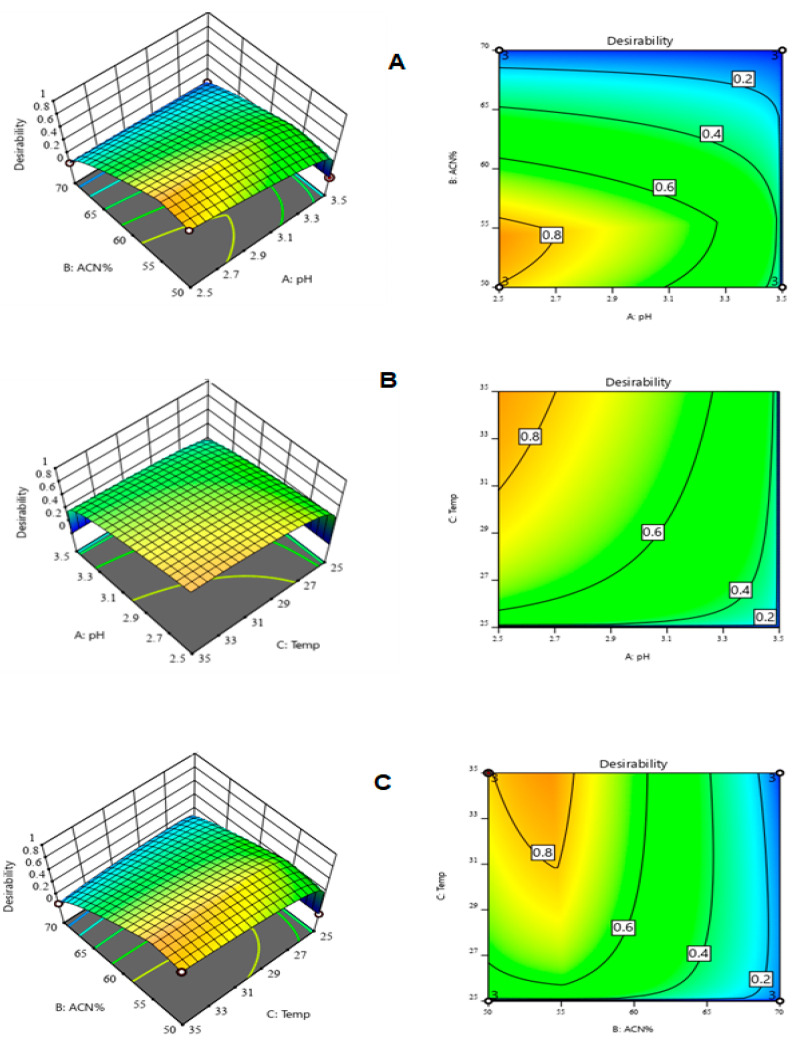
A 3D plot of Derringer’s desirability function in correlation with variable acetonitrile and pH (**A**), pH and temperature of the separation column (**B**), and temperature and acetonitrile composition % (**C**).

For better visualization of the results (Table 3), a 3D plot of the overall desirability function is presented in Figure 3.

### 3.4. Method’s Critical Parameters and Design Space

**Figure 4:**
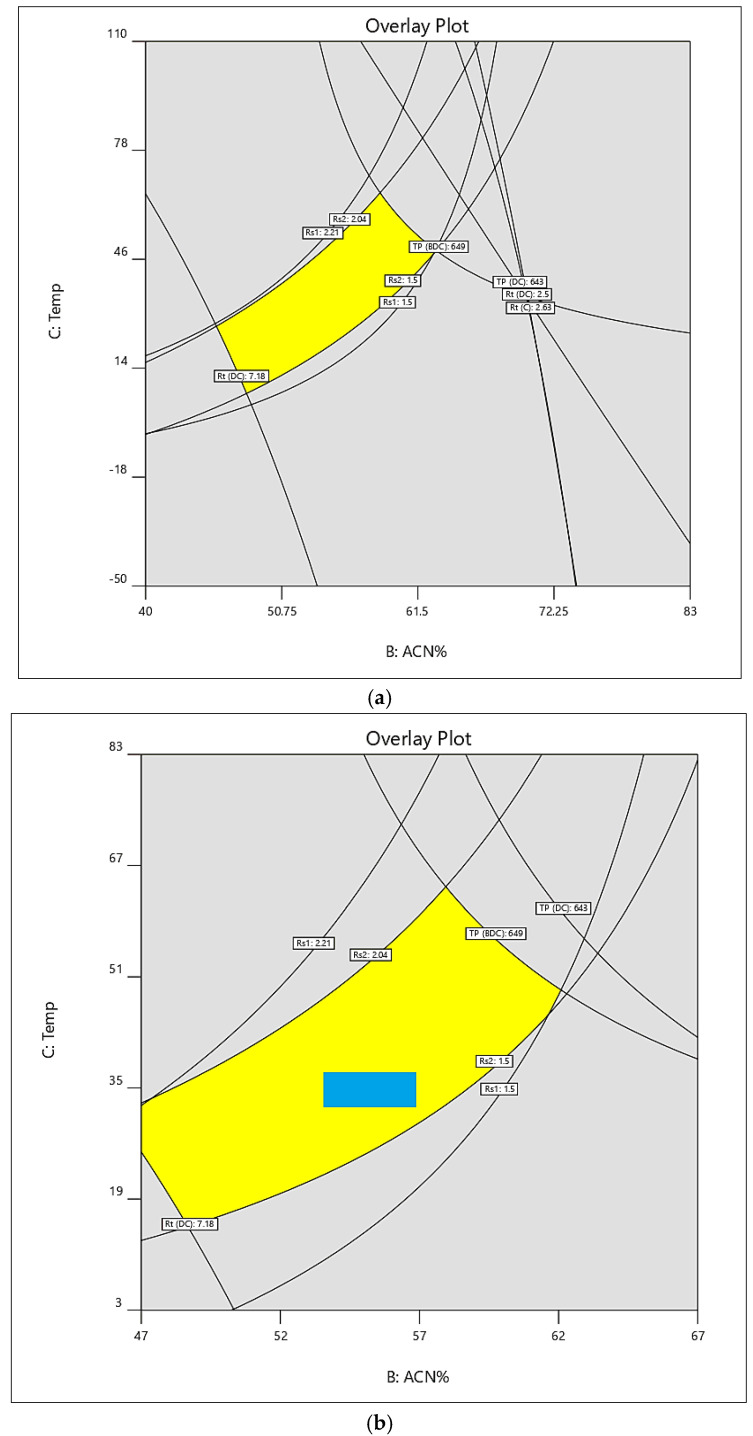
(**a**) Design space overlay incorporating the CMPs. (**b**) Design space generated for robust chromatography for curcuminoids.

An optimized value for the CMPs was established. A design space (Figure 4a) was generated that overlaid all the contours of the predicted as well as the acceptable limits within the design range and the allowed limits. The yellow region refers to the area in which all the CMPs were bound within the designed and predicted values of the parameters.

**Figure 5:**
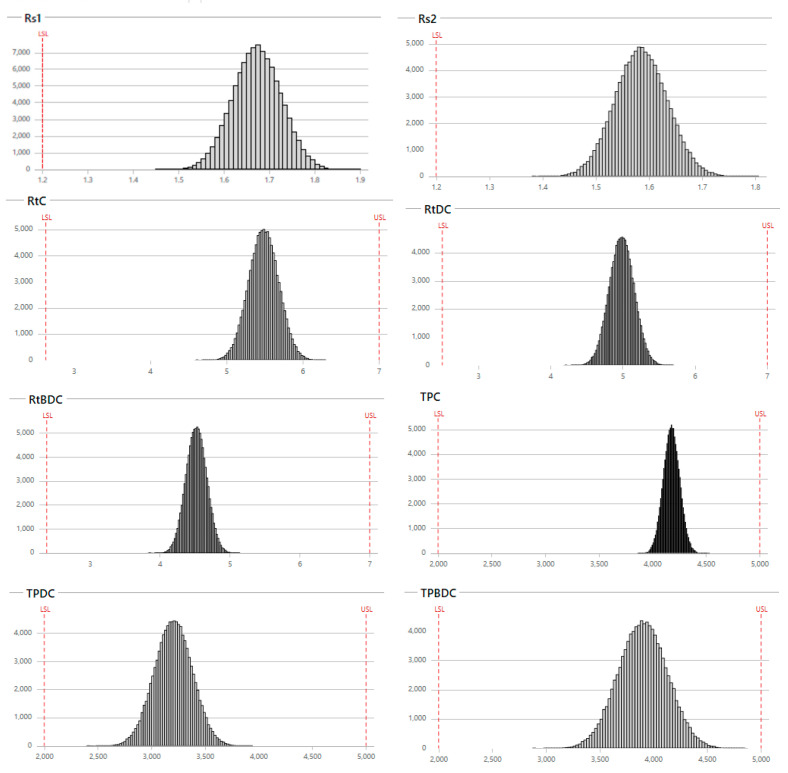
Monte Carlo simulation histograms of CMAs, and Cpk values with upper (right side row), and lower (left side row) specifications limits. The resolutions (Rs), retention time (RT), and theoretical plates (TP) were simulated for the curcumin (C), bisdemethoxycurcumin (BD), and desmethoxycurcumin (DC).

The optimized design space overlaid (yellow) area (Figure 4a) was selected, corresponding to intersects, to choose the method’s operable design’s region (MODR), also defined by pH (2.5–2.9), temperature (32–35 °C), and the acetonitrile composition (54–57%) as represented in the blue box (Figure 4b. Monte Carlo simulations (Table 4) for the choice of the parameters validated these inputs, wherein the predictive errors were computed through the probability to reach the desired objectives of the experimental levels of the CMPs, as well as the accepted limits of the method’s attributes by evaluating the capacity index (Cpk) value of the process (Figure 5).

### 3.5. Monte Carlo Simulations, Plackett–Burman Design, Robustness, LOD, and LOQ

**Figure 6:**
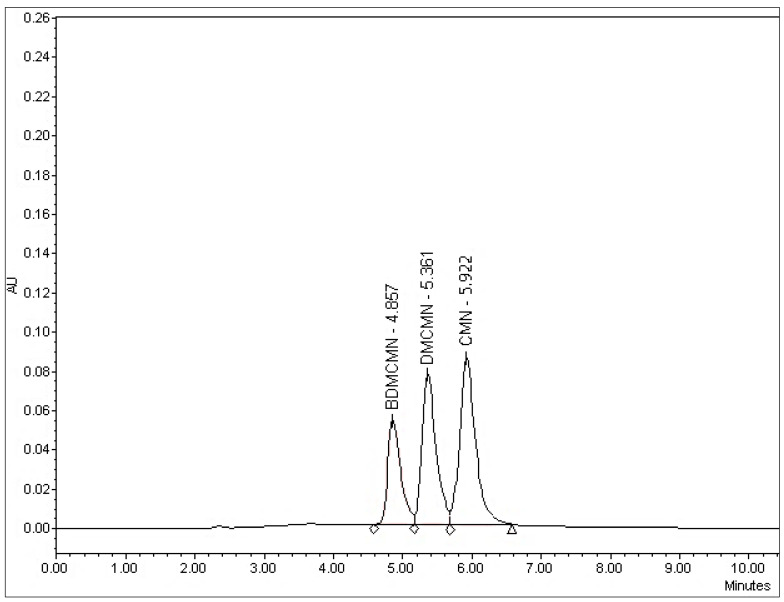
HPLC chromatogram obtained under the optimum conditions.

The MODR is considered an area of robustness (Figure 4b), wherein each point can be chosen as a working point; however, the practical considerations are of utmost priority as they fall within the common area between MODR and the designated experimental condition decided based on these designs encompassing the MODR. Some verification points (test points) were chosen before selecting a working point to verify the prediction. The practicability of the parameters was established from the obtained specifications within the design space limitations. The choices were guided through the temperature gradient feasible to be practically adopted later. In this overall context, the optimum conditions were selected whereby the mobile phase was composed of 54% acetonitrile (46% water) containing 10 mM phosphate buffer, pH at 2.7, and the column temperature at 33 °C. Figure 6 showed the experimental result of the RP-HPLC run (full-time run, 20 minutes chromatogram, view available in Supplementary File), and the resultant chromatogram under these designed, simulated, and validated conditions.

The robustness of the experimental method was again tested by employing the Plackett–Burman design (Table 5), wherein the effectiveness of the four critical method parameters was crisscrossed on eight critical method attributes. The selected parameters, i.e., the pH of the mobile phase (2.5–2.9), the ACN percentage (54–57%), column temperature (32–35 °C), and the buffer concentrations (8–12 mM), showed that in all the experiments, the effects of small changes in all of these parameters were producing insignificant effects (*p*-value > 0.05), and therefore, the method was considered robust.

The accuracy and precision of the designed and tested method were experimentally evaluated in the HPLC runs by injecting different concentrations of the curcumin (1, 10, 20 µg/mL), desmethoxycurcumin (1, 5, 9 µg/mL), and bisdemethoxycurcumin (1, 2, 4 µg/mL) in triplicate. The averaged area was determined, and the % recoveries and % RSD were calculated (Table S3).

The obtained % of RSD was less than two, thus indicating a good precision of the method. The good recoveries (98.76–99.89%) from the method also suggested an excellent accuracy of the developed method. The linearity of the developed method was established by analyzing the standard solutions, also in triplicate, of curcumin, demethoxycurcumin, and bisdemethoxycurcumin. Regression analysis showed good linearity, as indicated by the correlation coefficient values (>0.999).

The LOD and LOQ were calculated as 3.3 σ/s for LOD and 10 σ/s for LOQ, where σ is the standard deviation of the response and s is the slope, determined from the corresponding calibration curve. From Table 6, the LOD was determined as 0.024, 0.0105, and 0.335 μg mL^−1^ for the curcumin, demethoxycurcumin, and bisdemethoxycurcumin, respectively, whereas LOQ was found to be 0.075, 0.319, and 1.015 μg mL^−1^ for curcumin, demethoxycurcumin, and bisdemethoxycurcumin, respectively. These values indicated the acceptable sensitivity of the method to analyze the curcumin, demethoxycurcumin, and bisdemethoxycurcumin.

### 3.6. Previous Works, Present Method, and Analysis of Samples

Moreover, the present work was also compared with previous reports. The comparisons concerning the stationary phase, retention time, elution mode, detection wavelength, and limit of detection were carried out. The analysis is presented in Table 7, which showed that most of the reported methods used 1.5–2.0 mL min^−1^ as flow rates and consumed moderate quantities of expensive HPLC-grade solvents.

The retention time of these methods ranged between 11 and 28 min [33,34,35,36,37,38,39,40,41,42,43]. In retrospect, these methods are expensive and time-consuming. Additionally, in most cases the LOD is not provided [44,45,46,47,48,49,50,51], probably, owing to the poor separation. In some cases, the peaks were also not well resolved [45,46,49,52,53,54,55] and the detection limits were unacceptably high. The separation time was 2.5 min when the UPLC system was used, but this system is expensive and not available in most of the labs. Additionally, in one method [51], the separation time is 3.0 min on the accucore© column at high column temperature (40 °C) with the use of a high percentage of organic solvent.

**Table 7:** A comparison between the reported methods of curcuminoids separation by HPLC with the present work.

No.	Column	R_T_	Elution Mode	Detection Wavelength	Drawbacks	Ref.
1.	Partisphere5 WCX	21.0	Isocratic	280 nm	High separation time; catalytic effect of stationary phase; forced degradation; photo-oxidative; no detection limit	[44]
2.	C18	14.0	Gradient	250, 425 nm	Carried at 48 °C; curcuminoids as one peak; no detection limit	[45]
3.	C18	7.0	Isocratic	425 nm	low resolution; no detection limit	[49]
4.	C18	10.0	Isocratic	420 nm	High separation time	[52]
5.	TSK-GEL, ODS 80Ts	10.0	Isocratic	405 nm	Detection limits not provided	[46]
6.	C18	3.0	Isocratic	420 nm	Poor base-line; overlapping peaks	[55]
7.	C18	15.0	Isocratic	425 nm	High separation time; high cost	[56]
8.	C18	28.0	Isocratic	Fluorescence	High separation time; high cost	[57]
9.	C18	2.5	Gradient	420 nm	Expensive instrument; gradient elution	[58]
10.	C18	11.0	Gradient	420 nm	Gradient elute; no detection limit	[47]
11.	C18	17.0	Gradient	426 nm	High separation time; no detection limit	[48]
12.	C18	12.0	Isocratic	425 nm	Curcuminoids as one peak	[53]
13.	C18	14.0	Gradient	244, 360 nm	Retention times too close; gradient elution; detection limits not given	[49]
14.	Chromolith	21.0	Isocratic	425 nm	High retention time; high cost; retention times too close	[59]
15.	C18	20.0	Isocratic	425 nm	High retention time; *Forced degradation studies: Acid/base/oxidation/thermal/photo*	[60]
16.	C18 and Accucore^®^	3.0	Isocratic	428 nm	The high percent of organic modifier, column’s high temp	[51]
17.	RP-phenyl	10.5	Isocratic	360 nm	High retention time	[53]
18.	C18	18.0	Gradient	420 nm	High retention time; complex gradient	[15]
19.	C18	8.0	Isocratic	370 nm	Only for CMN	[45]
20.	C18	16.0	Isocratic	420 nm	High retention time; high cost; no stability studies	[61]
21.	Fused C18	3.0	Gradient	240-600	Complex gradient; no stability studies	[62]
22.	C18	24.5	Isocratic	425 nm	High retention time; high cost	[63]
23.	C18	11.0	Gradient	425 nm	High retention time; high cost; complex gradient	[10]
24.	C18	12.0	Isocratic	425 nm	High retention time; high column temp	[64]
25.	C18	8.0	Isocratic	425 nm	Long time for column conditioning and baseline stability; curcuminoids as one peak; *Degradation studies: Acid/base/oxidation/thermal/ photo*	[54]
26.	C18	3.0	Gradient	425 nm	Complex gradient; high column temp	[11]
27.	C18	12.0	Gradient	420 nm	High retention time; complex gradient	[14]

On the other hand, the current method is stability indicating, which showed sharp peaks with good resolution, although, for certain analyses, the resolution was less clear, probably owing to the column’s stationary phase materials’ physico-chemical conditions, which are not a contributor from the Q*b*D approach, and different factors considered, including the mobile phase characteristics. Additionally, the current approach needed a short experimental time and provided a new detection wavelength. The implementation through concepts of the Q*b*D approach made the current method novel, as well as owing to its tested workability based on design concepts through Method Operable Design Region (MODR) analysis.

The pharmaceutical dosage forms of the curcumin formulations were also tested for their chromatographic behavior. Solgar^®^ (full spectrum soft gel curcumin capsules × 5) containing 48 mg of curcuminoids, and Longvida^®^ (optimized curcumin tablets × 5) containing 1 g of turmeric powder were analyzed and compared with the ethanol and acetone extracts of the commercially available Solgar® product. A comparative analysis of the Solgar^®^ product, and acetone and ethanol extracts of the commercial powder showed higher RT respectively at 5.90 and 5.80 minutes for the curcumin, and RT for demethoxycurcumin and bisdemethoxycurcumin was observed at 5.37 and 4.86 min., respectively, for the Solgar®, and acetone and ethanol extracts of the commercial powder. The componential weight ratio of the Solgar® product was lesser than the commercial powder compounds, demethoxycurcumin, and bisdemethoxycurcumin (Figure S1). The acetone and ethanol extractions showed no variation in the contents ratio. A comparison of the currently developed and reported methods of analytical and viability studies is presented in Table 8 for further comparison.

An absence of new peaks in the pharmaceutical dosage forms chromatograms indicated that no interference was present from the additives and excipients of the formulation. As observed, the quantities of the three curcuminoids were variable, but in the same trend of occurrence as the curcumin, desmethoxycurcumin, and bisdemethoxycurcumin, respectively, in all the samples wherein the contents were found present in the ratio of curcumin > demethoxycurcumin > bisdemethoxycurcumin, as also present in the commercial curcumin powder extracts by the acetone and ethanol and the Solgar® sample. The comparison further validated the method’s reproducibility and non-interference of the capsules and tablets’ additives and excipients present in the formulations.

**Figure 7:**
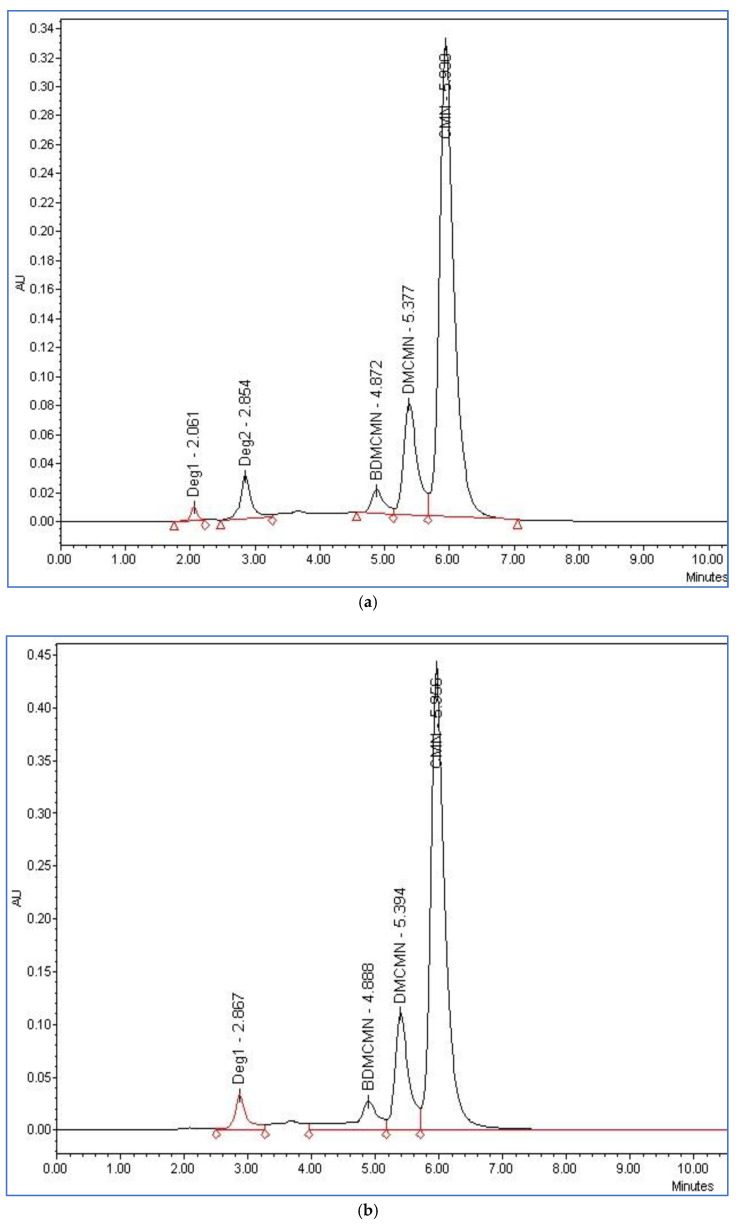

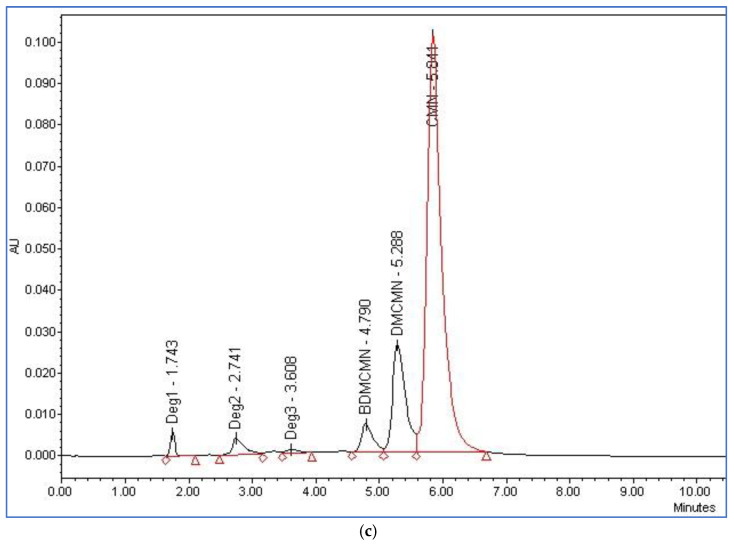
HPLC chromatograms of curcuminoids, (**a**) after exposure to acid degradation; (**b**) after exposure to base degradation; (**c**) after exposure to thermal degradation.

The forced-degradation studies were also carried out on the curcumin powder’s extract to produce the possible degradants and test their chromatographic behavior using the developed HPLC method in a simultaneous run of the method, and check its workability under the presence of other degradant materials obtained through various degrading conditions. The acid-induced degradants (Figure 7a) showed small-scale degradation (4.92%) with degradation peaks at 2.06 and 2.85 min, while the base-catalyzed degradation products were represented in Figure 7b, wherein a high level of degradation (79%) was observed, which also produced a new peak at 2.86 min.

**Figure 8:**
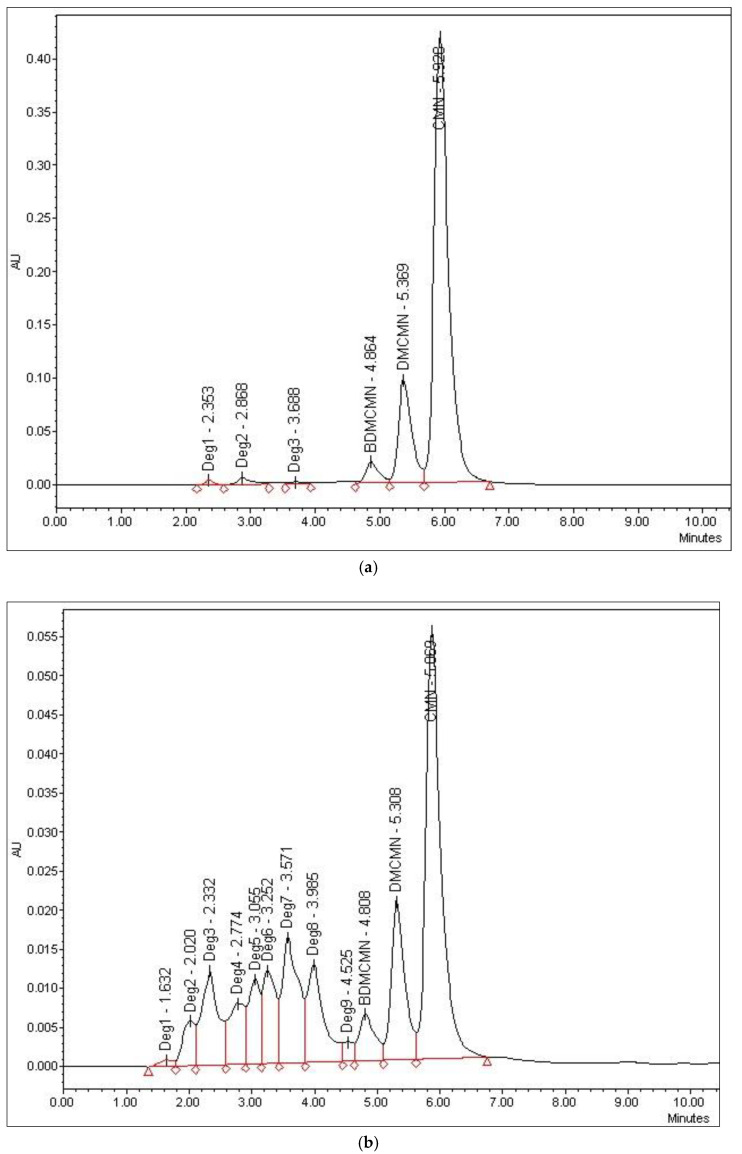

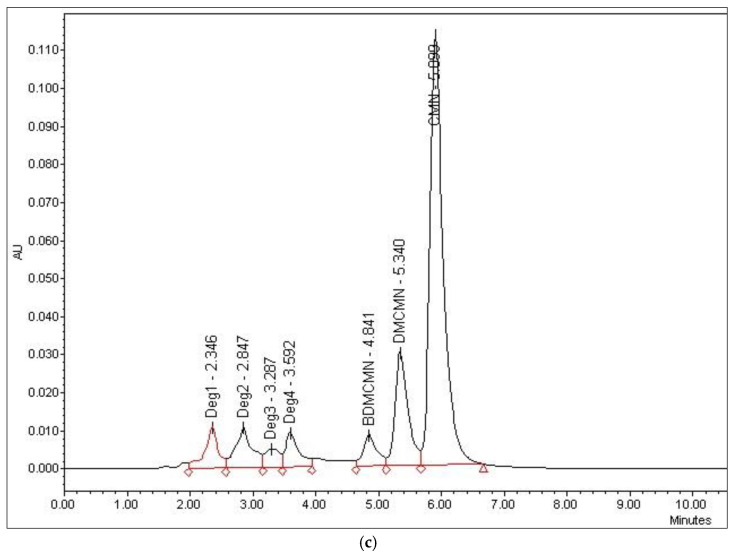
HPLC chromatograms of curcuminoids after exposure to (**a**) UV-irradiation; (**b**) degradation after exposure to sunlight; (**c**) degradation after exposure to oxidative conditions.

The thermal degradation of curcumin showed much less degradation (4.21%) and produced new peaks showing up at 1.74–3.60 min retention time in the chromatogram (Figure 7c). Under sunlight, a high degradation of curcuminoids was observed (47.2%), while oxidative conditions upon reacting with H_2_O_2_ produced 11.16% of degradation that were detected between 2.34 and 3.59 min of retention times. The UV-irradiation-based degradation was the lowest (Figure 8).

The degradation of curcuminoids under different stress conditions was evaluated to develop a stability-indicating method for the simultaneous determination of the curcuminoids in the *Curcuma longa* extract and pharmaceutical dosage forms. The acidic hydrolysis of the curcuminoids showed a small degradation (4.92%), and the degradation peaks were observed at 2.06 and 2.85 min. However, the alkaline hydrolysis of the curcuminoids showed higher degradation (21.45%), thereby producing a new peak showing at 2.86 min in the chromatogram of the degraded products with an area almost identical to the peak that appeared at its specific retention time (Figure 7). The oxidative degradation studies with H_2_O_2_ showed mild degradation of curcuminoids (11.16%), whereby the four degradation product peaks were observed between 2.34 and 3.59 min. Under irradiation at 254 nm, the HPLC chromatogram displayed a small-scale degradation of curcuminoids (2.6%) with the appearance of three degradation peaks ranging between 2.36 and 3.86 min (Figure 8). For thermal degradation, small degradation (4.21%) was observed and the peaks were seen between 1.74 and 3.60 min. The highest degradation (47.2%) was observed under the sunlight.

## 4. Conclusions

The Q*b*D approach was used to develop an HPLC-based detection and quantitation of curcuminoids and their forced-degradation products in a commercially available curcumin powder. The method was advanced on inputs from the requisites of the separation parameters of the products that relied on the critical attributes of resolutions of peaks in the chromatogram, retention times of the products and degradants. It also estimated the requirements of the theoretical plates for each constituent’s separation, suitable pH, and composition of the mobile phase, the composition of the mobile phase solvents, together with the temperature of the column during separation. The method was found to be robust in analysis, and the Monte Carlo simulations predicted the feasibility of the operational domains of the method’s factors-based values for experimental purposes, which itself was validated in several of the laboratory runs of the curcuminoid products and its degraded materials. The generated data, parametric perturbations, operational region’s predicted values and the products analyses unequivocally verified that the developed method was specific for the determination of curcuminoids, and is free from the interference of degradation products or overlapping of the peaks in the chromatogram. However, the overlapping is caused by column void and physical conditions of the stationary phase. The resolution was for the most part was clear, and the method was found to be stable in commercial products, capsules, tablets, and curcumin powder extracts analyses. The total separation time was less than 7.0 min, which contributed to the increased productivity of the method in comparison to the previously reported methods for the products. The Q*b*D approach helped to identify the sources of the method’s variability. It also helped in controlling the risk parameters to design a better, stable, and robust method, thereby extending the lifecycle of the currently developed analytical method, which also provided simultaneous separations of the curcuminoid products and their degradants under various conditions. The Q*b*D approach also enhanced the method development capability and reduced the time and operational cost of the chromatographic process. It also increased the safety of the analysis. This approach is also known as Analytical Quality by Design (AQ*b*D) and can be developed for other mixtures and pharmaceutical products of nutraceutical and extract-based origins. Moreover, the current method development provides an approach for the analysis of food components, other natural as well as synthetic drugs and their impurity profiling, and degradants analysis, including the analysis in clinical settings. The approach can also help to purify, identify, compare, and quantify, as well as bulk isolate, the principal and individual components of a mixture in a robust and feasible manner. The method also laid down the approach details towards a designated time-defined optimum resolution for complex and labile mixtures, a comparison-based validation of the componential analysis, and the resolution of light, heat and pH sensitive materials. The current method developmental approach, and the method itself may find broader applicability for the analytical chemist, clinical and forensic analyst, and analytical toxicologist, as the case and requirements may be. Apart from clinical studies involving HPLC, medico-legal comparatives for components and techniques involving competitive protein binding assays and HPLC comparisons for sensitivity, and LOQ and LOD-based analysis, immunoassays confirmation and comparisons of material analysis through HPLC are of importance and various comparisons with other techniques for purification and componential analysis techniques and parables have been undertaken in the past. This method may provide a better HPLC based analysis based on Q*b*D approach. Not to mention, the accuracy of the HPLC chromatographic technique is still of unparalleled importance for pharmaceutical, and other genres of analytical chemists, and the approach can serve to show the prime importance of the singular method of analysis, the HPLC/RP-HPLC, for purity and contents qualitative and quantitative analysis.

## Figures and Tables

**Table 1:** The selected factors and their levels.

Chromatographic Factors	Levels Used in the Experiments		
	Low	Medium	High
pH	2.5	3	3.5
% ACN	50	60	70
Temperature (° C)	25	30	35

**Table 2:** The statistical summary of all the responses.

Response	F	Model *p*-Value	% CV	R^2^ (R-Squared)	Adj R^2^	Adeq. Precision
RS_1_	344.27	<0.0001	3.76	0.992	0.989	39.93
RS_2_	651.13	<0.0001	2.51	0.994	0.991	59.83
RT_CMN_	316.27	<0.0001	4.79	0.991	0.988	36.76
RT_DMCMN_	410.47	<0.0001	4.05	0.993	0.991	42.37
RT_Bisdemethoxycurcumin_	397.74	<0.0001	3.89	0.993	0.991	42.25
TP_CMN_	315.96	<0.0001	2.6	0.991	0.988	47.00
TP_DMCMN_	196.14	<0.0001	8.22	0.987	0.982	34.37
TP_Bisdemethoxycurcmin_	662.65	<0.0001	4.87	0.996	0.994	56.61

RS stands for Resolution, RT stands for Retention Time, and TP stands for Theoretical Plates.

**Table 3:** The optimization standards for each CMA.

CMAs	Upper Limit	Lower Limit	Goal	Results	
				Predicted	Experimental
Resolution between curcumin and demethoxycurcumin (RS1)	2.12	0.85	>1.2	1.87	2.05
Resolution between demethoxycurcumin and bisdemethoxycurcumin (RS2)	2.04	0.83	>1.2	1.77	1.88
Curcumin RT (minutes)	6.51	2.38	Maximize	4.65	4.86
Demethoxycurcumin RT (min.)	7.18	2.51	In range	5.47	5.36
Bisdemethoxycurcumin RT (min.)	7.9	2.66	Minimize	6.02	5.93
Number of theoretical plates of curcumin	5800	655	>2000	4818	5122
Number of theoretical plates of demethoxycurcumin	4644	679	>2000	3937	4144
Number of theoretical plates of bisdemethoxycurcumin	3699	2637	>2000	4905	5249

**Table 4:** CMPs values obtained by the Monte Carlo simulation method and the predicted separation model’s summary.

No.	CMPs	Distribution Type		CMPs Range		CMAs	Specification Limits	
				Low	High		Low	High
1.	Acetonitrile composition (%)	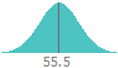		54	57	RS1	1.2	12
2.	pH	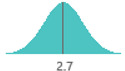		2.5	2.9	RS2	1.2	12
3.	Temperature	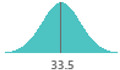		32	35	R_T_ Curcumin	2 (min)	7 (min)
						R_T_ Demethoxycurcumin	2 (min)	7 (min)

						R_T_ Bisdemethoxy curcumin	2 (min)	7 (min)
						TP Curcumin	2000	≥2000
						TP Demethoxy curcumin	2000	≥2000
						TP Bisdemethoxy curcumin	2000	≥2000
Predicted model’s summary				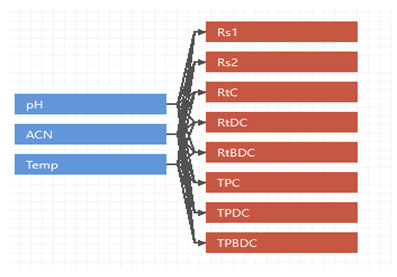				

**Table 5:** The experimental plan and responses of the Plackett–Burman design for robustness test.

Run	CMPs Variations				CMAs Responses							
	1	2	3	4	1	2	3	4	5	6	7	8
	pH	Acetonitrile %	Temperature °C	Buffer mM	Resolution 1	Resolution 2	R_T_(BDMCR)	R_T_ (DMCR)	R_T_(CR)	TP (BDMCR)	TP (DMCR)	TP (CR)
*1*	*2.5*	*54*	*35*	*8*	*1.84*	*1.729*	*4.878*	*5.373*	*5.919*	*4806*	*3970*	*4915*
*2*	*2.9*	*54*	*35*	*12*	*1.84*	*1.728*	*4.88*	*5.374*	*5.923*	*4808*	*3966*	*4919*
*3*	*2.9*	*54*	*35*	*12*	*1.84*	*1.728*	*4.88*	*5.374*	*5.924*	*4809*	*3965*	*4920*
*4*	*2.9*	*57*	*32*	*8*	*1.834*	*1.719*	*4.877*	*5.368*	*5.918*	*4805*	*3968*	*4918*
*5*	*2.9*	*57*	*35*	*8*	*1.836*	*1.722*	*4.875*	*5.369*	*5.917*	*4801*	*3965*	*4911*
*6*	*2.5*	*54*	*32*	*8*	*1.838*	*1.726*	*4.88*	*5.374*	*5.92*	*4810*	*3973*	*4920*
*7*	*2.5*	*57*	*32*	*12*	*1.836*	*1.721*	*4.877*	*5.369*	*5.916*	*4805*	*3967*	*4909*
*8*	*2.5*	*54*	*32*	*12*	*1.839*	*1.726*	*4.881*	*5.374*	*5.921*	*4811*	*3974*	*4917*
*9*	*2.5*	*57*	*35*	*8*	*1.837*	*1.724*	*4.874*	*5.368*	*5.914*	*4800*	*3964*	*4908*
*10*	*2.9*	*57*	*32*	*12*	*1.835*	*1.72*	*4.878*	*5.37*	*5.919*	*4806*	*3969*	*4913*
*11*	*2.9*	*54*	*32*	*8*	*1.836*	*1.724*	*4.881*	*5.375*	*5.923*	*4811*	*3968*	*4919*
*12*	*2.5*	*57*	*35*	*12*	*1.838*	*1.725*	*4.875*	*5.368*	*5.915*	*4801*	*3965*	*4909*
*13*	*2.7*	*55.5*	*33.5*	*10*	*1.836*	*1.721*	*4.874*	*5.374*	*5.915*	*4813*	*3977*	*4916*
*14*	*2.7*	*55.5*	*33.5*	*10*	*1.833*	*1.726*	*4.879*	*5.369*	*5.921*	*4807*	*3971*	*4923*

**Table 6:** Statistical parameters for individual calibration curves.

Parameters	Curcumin	Demethoxycurcumin	Bisdemethoxycurcumin
Linearity (µg/mL)	0.76–24.5	0.51–10.0	0.13–4.3
Slope	2,222,250	345,7396	1,077,486.9
Intercept	499,471.1	−414,324.79	−133,404.51
R^2^	0.9997	0.9997	0.9986
LOD	0.024	0.0105	0.335
LOQ	0.075	0.319	1.015

**Table 8:** Application of the proposed HPLC method for the analysis of the contents of curcuminoids (µg/mL) in *Curcuma longa* powder extract and pharmaceutical dosage forms.

Source/Dosage Form	API	Content (mg/gm) ± S.D.		*t-*Value *	F-Value *
		Present Method (*n* = 3)	Reported Method [36], (*n* = 3)		
*Solgar^®^ Full Spectrum* *capsules*	Curcumin	27.15 ± 0.35	27.62 ± 0.26	1.87	1.76
	Demethoxycurcumin	15.6 ± 0.24	15.86 ± 0.15	1.02	2.67
	Bisdemethoxycurcumin	3.78 ± 0.17	3.80 ± 0.18	0.11	1.05

*Longvida^®^ Optimized Curcumin* *tablets*	Curcumin	21.84 ± 0.03	21.86 ± 0.08	0.309	5.37
	Demethoxycurcumin	13.6 ± 0.12	13.62 ± 0.11	0.241	1.11
	Bisdemethoxycurcumin	3.36 ± 0.07	3.36 ± 0.06	0.341	1.31

*Ethanol extracts*	Curcumin	31.71 ± 0.60	31.54 ± 0.09	2.62	1.83
	Demethoxycurcumin	9.81 ± 0.13	9.84 ± 0.14	0.322	1.21
	Bisdemethoxycurcumin	3.84 ± 0.15	3.77 ± 0.25	0.372	2.59

*Acetone extracts*	Curcumin	20.28 ± 0.17	20.40 ± 0.44	0.454	6.37
	Demethoxycurcumin	8.37 ± 0.15	8.52 ± 0.36	0.659	5.09
	Bisdemethoxycurcumin	3.97 ± 0.12	3.96 ± 0.10	0.175	1.31

* Tabulated values at 95% confidence limit, t = 2.78 and F = 6.39.

## Data Availability

Data are contained in the manuscript and are provided in the Supplementary File.

## Supplementary Materials

The following supporting information can be downloaded at: https://www.mdpi.com/article/10.3390/foods12051010/s1, Table S1: Design matrix with factorials and responses; Table S2: Model equations for all other responses; Model equations for all other responses; Table S3: Intraday and interday accuracy and precision of curcuminoids; Figure S1: Full time (20 min.) HPLC chromatogram obtained under the optimum conditions of Solgar® Curcumin Full Spectrum (A); Curcuma longa after extraction with acetone (B); Curcuma longa after extraction with ethanol (C).
