# Supplementary material for: Analytical Purity Determinations of Universal Food-Spice Curcuma longa through a QbD Validated HPLC Approach with Critical Parametric Predictors and Operable-Design’s Monte Carlo Simulations: Analysis of Extracts, Forced-Degradants, and Capsules and Tablets-Based Pharmaceutical Dosage Forms
Source: Foods. 2023 Feb 27;12(5):1010. doi: 10.3390/foods12051010 (PMC10000774; doi:10.3390/foods12051010)
Supplement: Supplementary file 1 [file foods-12-01010-s001.zip › foods-2069488-supplementary.pdf]

<u>*Supplementary File*</u>

Analytical Purity Determinations of Universal Food-Spice *Curcuma longa* through a Q*b*D Validated HPLC Approach with Critical Parametric Predictors and Operable-Design's Monte Carlo Simulations: Analysis of Extracts, Forced-Degradants, and Capsules and Tablets-Based Pharmaceutical Dosage Forms

<u>**TABLES:**</u> **Table S1.** Design matrix with factorials and responses

Run	Factor			Response							
	1	2	3	1	2	3	4	5	6	7	8
	A: pH	B: ACN (%)	C: Temp .(ºC)	Resolution 1	Resolution 2	RT BDMCR)	RT (DMCR)	RT (CR)	TP BDMCR)	TP (DMCR)	TP (CR)
1	3.5	50	35	1.98	1.78	5.82	6.52	7.22	4795	3708	3747
2	2.5	50	35	2.12	2	5.39	6	6.66	5893	4827	5331
3	3	60	30	1.42	1.36	4.22	4.3	4.53	4753	3344	3637
4	2.5	50	25	1.85	1.75	5.49	6.02	6.59	5971	5019	5345
5	3.5	70	35	0.89	0.9	2.38	2.51	2.66	655	679	2637
6	2.5	70	25	0.98	0.85	2.51	2.64	2.79	666	685	3523
7	3.5	50	35	2	1.89	5.87	6.55	7.29	5463	4686	3937
8	2.5	70	35	0.93	0.94	2.38	2.51	2.66	687	643	3430
9	3.5	50	25	1.98	1.53	6.51	7.18	7.9	5800	4644	3699
10	2.5	70	35	0.92	0.93	2.39	2.51	2.66	679	655	3430
11	2.5	70	25	0.85	0.83	2.45	2.57	2.72	675	1139	3389
12	3.5	50	35	2	1.89	5.87	6.55	7.29	5012	3879	3823
13	3.5	70	25	0.9	0.92	2.41	2.5	2.63	2911	2639	2888
14	2.5	50	35	2.1	1.97	5.39	5.99	6.64	5869	4738	5204
15	3.5	50	25	1.86	1.55	6.48	7.14	7.83	5659	3262	3727
16	2.5	70	25	0.848	0.832	2.51	2.64	2.79	6777	1190	3523
17	3	60	30	1.44	1.34	4.18	4.32	4.48	4813	3042	3631
18	2.5	50	35	2.21	2.04	5.84	6.5	7.21	6134	4986	5456
19	2.5	50	25	2.02	1.87	6.04	6.65	7.31	6428	5600	5683
20	2.5	70	35	0.94	0.95	2.39	2.51	2.66	687	645	3268
21	3	60	30	1.43	1.31	4.21	4.39	5.1	4855	3265	3635
22	3.5	70	25	0.9	0.91	2.44	2.56	2.7	2895	2652	2892
23	3.5	70	35	0.9	0.92	2.38	2.51	2.66	655	684	2876
24	3.5	50	25	1.79	1.54	5.99	6.6	7.23	5290	3242	3625

25	*2.5*	*50*	*25*	*1.9*	*1.78*	*5.88*	*6.46*	*7.07*	*6227*	*5036*	*5463*
26	*3.5*	*70*	*35*	*0.91*	*0.91*	*2.38*	*2.51*	*2.66*	*649*	*695*	*2856*
27	*3.5*	*70*	*25*	*0.91*	*0.92*	*2.42*	*2.55*	*2.69*	*2881*	*2599*	*2895*

Table S2. Model equations for all other responses

Response	Model
R_{s1}	=+1.65833+0.46pH -0.016283ACN +0.132667Temp -0.004967pH*ACN -0.026pH*Temp -0.001697ACN * Temp +0.000313pH*ACN*Temp
R_{s2}	=+9.95458-2.75417pH-0.146508ACN-0.119083Temp+0.044383pH*ACN+0.065833pH *Temp+0.002238ACN*Temp-0.001097pH *ACN* Temp
(Rt_{CMN})	=+5.27333+5.18pH-0.020333ACN+0.2355Temp-0.078667pH*ACN-0.105pH * Temp-0.003850ACN * Temp +0.001633pH * ACN * Temp
(Rt_{DMCMN})	=+5.19333+4.71333pH-0.023ACN+0.1945Temp-0.071333pH*ACN-0.097pH* Temp-0.003217ACN * Temp+0.0015pH*ACN*Temp
(Rt_{BDMCMN})	=+5.21792+4.21083pH-0.027542ACN+0.154917Temp-0.06325pH*ACN-0.0885pH * Temp-0.002575ACN * Temp+0.00135pH * ACN * Temp
(TP_{CMN})	=+30528.04167-7666.25pH-361.7875ACN-311.375Temp+101.125pH * ACN+111.45pH*Temp+4.30083 ACN*Temp-1.59167pH*ACN*Temp
(TP_{DMCMN})	=+95386.66-31458.16 pH-1535.3ACN-2089.88Temp+529.3pH*ACN +820.13pH * Temp+35.01ACN * Temp-13.98pH * ACN * Temp
(TP_{BDMCMN})	=+71381.58-19652.33 pH-1291.21ACN-1278.58Temp+393.01pH* ACN+476.2pH * Temp+26.33ACN * Temp-10.02pH * ACN * Temp

Table S3. Intraday and interday accuracy and precision of curcuminoids

Compound	Concentration	Intraday		Interday	
		Accuracy (recovery%± SD)	Repeatability (RSD%)	Accuracy (recovery%± SD)	Repeatability (RSD%)
CMN	1	99.63±0.37	0.38	99.32±0.34	0.35
	10	99.65±0.41	0.41	99.56±0.39	0.4
	20	98.76±0.32	0.32	99.51±0.17	0.17
DMCMN	1	99.13±0.7	0.71	99.29±0.64	0.65
	5	99.6±0.4	0.41	99.53±0.5	0.51
	9	99.89±0.63	0.64	99.71±0.26	0.27
BDMCMN	1	99.73±1.66	1.67	99.84±0.19	0.19
	2	99.71±0.59	0.6	99.81±0.09	0.09
	4	99.89±0.63	0.64	99.43±0.74	0.75

FIGURES:

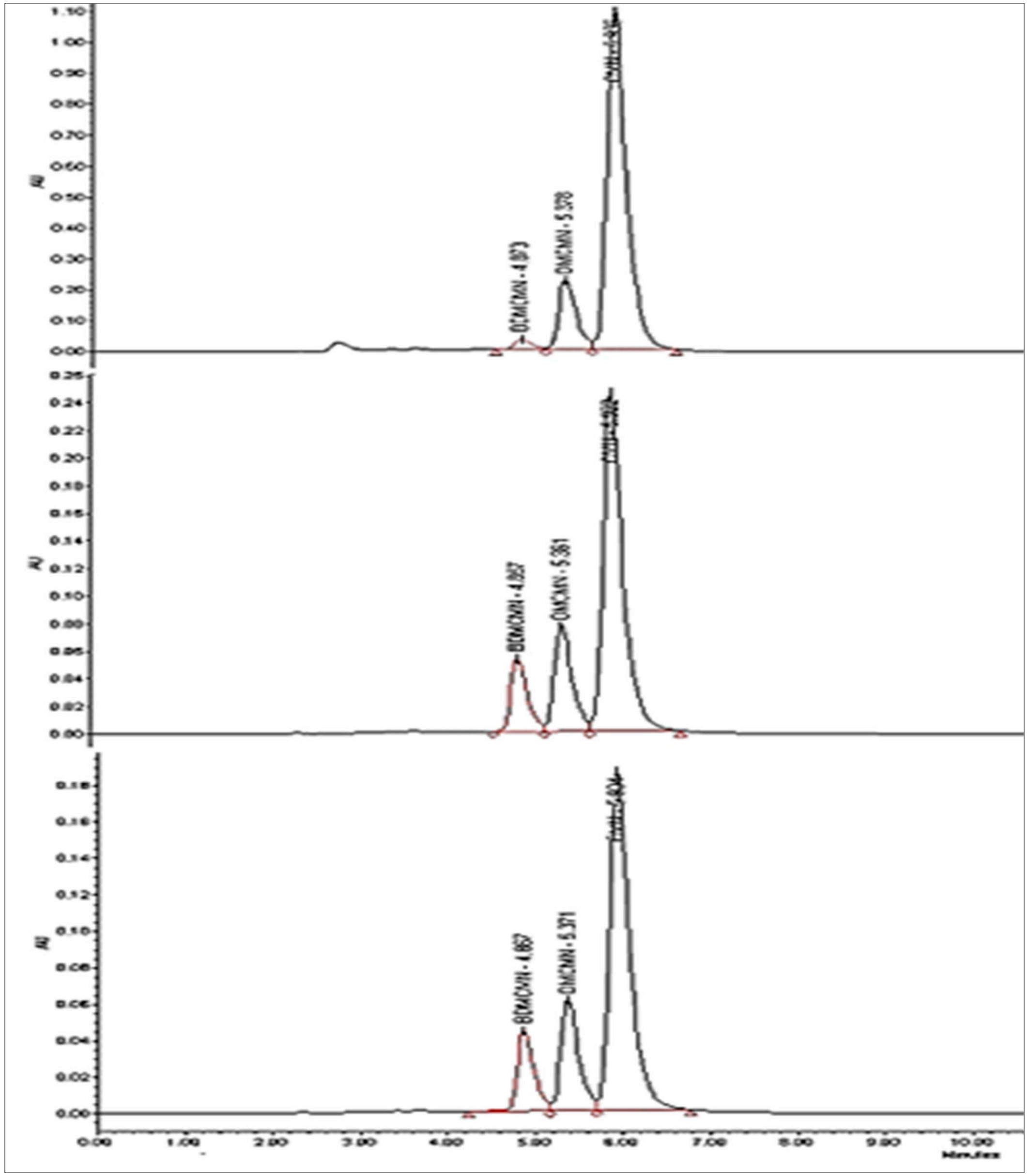

Figure S1. Full time (20 min.) HPLC chromatogram obtained under the optimum conditions of Solgar® Curcumin Full Spectrum (A); *Curcuma longa* after extraction with acetone (B); *Curcuma longa* after extraction with ethanol (C).
